# Therapeutics of the Throat: Belladonna

**Published:** 1887-11

**Authors:** W. S. Gee

**Affiliations:** Chicago, Ill.


### BELLADONNA.

W. S. Gee, M. D., Chicago, Ill.

*Objective.* Dryness of the mouth and throat. Mucous membrane reddened. Bright red, later dark red. Looks very red and shining. Tonsils swollen on right side, with intense congestion. Rapidly forming ulcers of an aphthous character on the tonsils. Fauces bright red or bright and yellowish red.

"Inflammation of the throat, with sensation of a lump, which induces hawking, with dark redness and swelling of the velum palate and pudendum."—*Lippe.* Catarrhal inflammation of the palate. Rawness and soreness of the palate. Tongue and palate dark-red.

The mucous membrane, from the posterior third of the palate as far down as could be seen, was of a deep crimson color, and the tonsils were much enlarged. The fauces, uvula, and tonsils are scarlet and shining. Throat swollen outside and sensitive to touch.

Cervical glands inflame suddenly. Right hand clutches at the throat. (Epilepsy.)

*Subjective.* 2. Dryness of roof of mouth, fauces, and throat. Difficulty of swallowing even liquids—they return through the nose.

Constant desire to swallow, with urging as if he would choke if he did not swallow.

During deglutition, feeling in the throat at if it were too narrow, or drawn together, as if nothing would pass properly. Desires drink but cannot swallow. Throat feels raw and sore.

Desires drink but is not satisfied with it. Desire to swallow saliva, which is painful.

About the fauces the sensation of dryness was most distressing; it induced a constant attempt at deglutition, and finally excited suffocative spasms of the fauces and glottis; renewed at every attempt to swallow.

The dryness causes alteration of voice, rawness and soreness of palate. Painful narrowing and contraction of the gullet.

Burning sensation in the fauces. Tonsillitis; worse right side; parts bright-red; worse swallowing liquids. Fine tearing on inner surface of angle of left lower jaw, in and behind left tonsil; unaffected by contact; the tearing is more violent during deglutition.

Violent burning in the throat (the mouth at the same time being moist) which is not relieved by drinking, but is by a little sugar, though only for a moment. Sensation as if a large tumor were growing in the throat and stopped it up. Speech thick. Shootings in pharynx; pain as from an internal swelling, only felt during deglutition and upon turning the head around; likewise when feeling side of neck, but not when at rest or in speaking.

Spasmodic constriction of the throat.

Only with difficulty and by constantly swallowing liquids is he able to swallow solid food.

On attempting to pour down liquid, tetanic closure of the mouth and regurgitation of liquid.

Constant desire for empty swallowing, which causes pain in larynx.

Swallowing saliva or empty is the most painful. Has to swallow, which is very painful; he has to bend head forward and lift up the knee. (Angina.) Throbbing of carotids.

Throat is painful to touch on right side, especially toward ear, where it stings.

Pressive pain externally when bending the head backward and when touching the parts.

Aggravation: from touch; from swallowing liquids or solids.

*Concomitants.* Flushed face, injected conjunctiva, throbbing headache.

Thirst for water, but especially lemonade, which agrees.

Dry cough, from dampness in larynx.

Swelling of glands in nape of neck, with cloudiness of head.

Pulse has a globular feel, and the skin feels very hot to touch. Sudden startings, especially on falling asleep.

Pains come and go suddenly.

Suits plethoric, lymphatic constitutions, who are jovial and entertaining when well, but violent when sick. Women, children, blue eyes, light hair, fine complexion, delicate skin.

Young, full-blooded; with fevers and inflammations when pulse and heat run high, congestions, nervous, delirious, threatened convulsions.

REMARKS.

*Differentiation.* Bell. is perhaps the remedy most frequently indicated for the acute anginas brought on by changes of the weather. It is not "the best remedy for sore throat," and should be given only when the symptoms of the patient indicate it.

The pernicious habit of giving this remedy in a routine way is hazardous to the patient and compromising, not to say slovenly, careless in the prescriber.

A study of the poisonous effects will show us first, *dryness* of mucous membrane of mouth, throat, even to stomach. *Burning* follows, spasmodic closure of glottis and œsophagus, so that we have "constant inclination to swallow, with feeling as though he would choke if he did not." This is characteristic of the remedy. Desire to swallow is occasioned by the dryness, and no relief from swallowing.

Recall another remedy of great value when the *dryness* is a pronounced symptom, viz.: Cist.-can., but the swallowing relieves and the throat is even *better after eating*.

The contracted, narrow feeling is similar to that of Caust., but the *dryness* will distinguish between them.

The "rapidly forming ulcers on tonsils" might remind us of Nit.-ac., but the pricking, splinter-like pain will decide for the latter remedy. Signs of ulceration of gums or throat will also be present, usually when Nit.-ac. is the remedy.

"Painful dryness, roughness, rawness, and burning in the throat" occurs in Merc. sol., but "with a mouth full of saliva."

Merc. cyan. has the small ulcers on the tonsils, but the symptom confirmed has been tonsils studded with small ulcers with coating, like drops of mortar in appearance.

This has been a reliable guide in many cases of serious throat trouble where the prostration was present (CM potency).

Sul. has "sensation of lump in the throat, stitches in throat when swallowing, painful contraction when swallowing, great burning and dryness, first right then left side," but this antipsoric is better suited to prolonged cases, for we have "whole back part posterior to palatine arches appears in a state of ulceration."

Lyc. includes "pharynx feels contracted; nothing can be swallowed; pain and soreness begin on right side of throat," but the changing to left side, and aggravated from cold drinks makes a distinction. The *course* of the disease is a more important indication for Lyc., Lach., Lac can., Sabad., and Sul. than for Belladonna.

---

Hering Memorial Hospital: a full account of the opening of this hospital is given in this issue; it is to be hoped that *all* admirers of Dr. Hering and all lovers of true Homœopathy will assist these ladies in the support and development of their noble work.

